# Trip and Trip Pro

**DOI:** 10.5195/jmla.2018.399

**Published:** 2018-04-01

**Authors:** Lynne M. Fox

**Affiliations:** Clinical Research Library, Saint Joseph Hospital, Denver, CO

## INTRODUCTION

The Trip database began more than twenty years ago in an effort to simplify searching for clinically relevant, high-quality, evidence-based information. Originally an acronym for Turning Research Into Practice, Trip is a meta-search tool and a convenient first stop for busy clinicians, librarians, and students who are seeking evidence for practice or learning. It is an excellent tool for librarians when they are developing search vocabulary during the early stages of a systematic review. Searching Trip can yield additional studies for systematic reviews because of its unique approach to retrieval.

A comparison chart clearly outlines the free versus fee features [1]. Trip Pro subscribers access greater content and more advanced search features. Free Trip users are prompted to consider Trip Pro when they attempt to access locked features so that they can evaluate whether a Pro subscription is warranted. An informal advisory group, composed of librarians and clinicians with database experience or content knowledge, provides guidance to Trip founder Jon Brassey about technical improvements or appropriate evidence-based content to add. They are sometimes asked to test prototypes of new features. The advisory group also helps Brassey better understand Trip’s users’ needs for evidence-based information, since he is neither a librarian nor clinician. The home page links to About, How to Use, and Liberating the Literature Blog. Tour links provide extensive information about using Trip and describe new features and proposed features that are currently being tested by the advisory group [2].

## USER ACCOUNT

Users of all types of accounts are encouraged to register. Accounts allow users to change their passwords or associated email addresses; select a clinical interest, profession, and affiliated institution to access full-text collections; set up customized search strategies or specialty alerts; mark articles as favorites; return to prior searches via a history list; and receive updates and alerts from Trip.

## SEARCHING AND CONTENT

Trip’s motto is “Find Evidence Fast.” Multiple search types and functions are available, including Boolean operators, asterisk truncation, phrase searching, proximity searching, and date and title limit searching. Trip Pro users can use basic or advanced search or combine search statements using the “Recent” search feature. A patient/problem, intervention, comparison, outcome (PICO) search form is available to assist with retrieving evidence. Search term suggestions, based on searches by other users, appear when a topic is typed into the search box. Spelling and typing errors are corrected by a polite, “search for…instead?” prompt. Another helpful crowdsourced prompt, “Trip users also search for,” aids users in finding associated search topics. For those searching multiple topics or trying multiple iterations of a single topic, a clear query box function would be helpful in the basic search.

The focus of Trip’s content is high-quality, evidence-based resources such as systematic reviews, clinical guidelines, and evidence-based synopses. These include a broad range of grey literature including regulatory advice, clinical trials, critically appraised topics (CATs), and patient decision aids. Trip users can access ten monographs per month from EBSCO DynaMed. Appropriate evidence-based resources are added at the suggestion of users or advisory group members, with a list of included publications provided [3, 4]. Trip includes open access evidence-based content from PubMed Central and other government, private, and foundation full-text. Trip’s PubMed content is updated every two weeks. Other Trip content is updated monthly [5]. Categories of evidence and the types of content that fit each category are neatly explained, using the evidence pyramid that is familiar to evidence-based practitioners.

## RETRIEVAL AND FILTERING

An algorithm scores the search results to determine their order and then sorts results based on quality of evidence. Users will find systematic reviews, primary research, evidence-based synopses, and clinical trials at the top of the results. A text score uses search term position and frequency; the publication score assigns a rank based on general quality of evidence for a publication; and the date score moves recent documents higher in the results. Filter options appear to the right of the results. Filters include evidence/publication types, search hedges for overdiagnosis and world development, limits for years, and links to previously viewed or starred favorites. A clinical filter allows users to limit results to a practice specialty. After selecting a result, a SmartSearch prompt appears offering closely related titles and other titles based on clickstream data [6].

## RESULTS DISPLAY

Default display is by quality. Users can also display results by date, relevance, and popularity based on user views. For example, the popularity display for *naloxone* includes recent hot topics such as guidelines addressing use of the drug in recovery rooms, end-of-life care, substance abuse, and patient-controlled analgesia. Crowdsourced data are also used in the “Latest & Greatest” feature, which provides a list of the most frequently visited results for a topic searched in the past twelve months [7]. As Trip’s user base continues to expand, crowdsourced features will become more powerful and useful.

**Figure 1:**
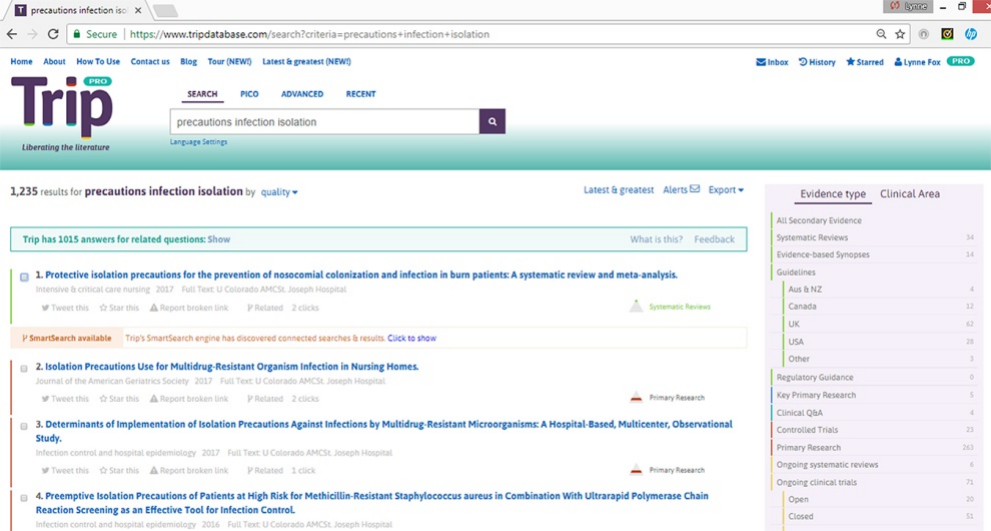
Sample Trip results

The results display includes a color bar that links a result to publication type filters (Figure 1). Other data in the display include the title, source and date, an OpenURL link if the user has configured one in their user profile, and links to tweet the citation, save the result in a user profile, report a broken link, and view related articles and number of times that the result has been viewed by other users. The results display also includes the publication type and an indicator of the source position in the evidence sources pyramid. The abstract cannot be displayed in the results list.

## FULL-TEXT

PubMed Central (PMC) and other Trip partner sites provide full-text, open access content through title links in the results list. Trip also offers OpenURL linking to hundreds of institutional full-text collections. Registered users select an institution (or multiple institutions if they are affiliated with more than one) in their user profiles. Institutional full-text links appear below results and may require institutional authentication for access. Librarians can send OpenURL linking information to Trip to be added as a full-text provider.

## EXPORT AND ALERTS

Trip Pro allows users to email results or export them in comma-separated values (CSV) and Research Information Systems (RIS) formats. Test files imported smoothly into Endnote Web, but users will need to supplement data in some references. Some non-journal resources have incomplete metadata, which results in incomplete citation data in RIS files.

Users can set up search topic alerts by performing a search, then clicking the Alerts link. Email alerts arrive monthly and include updates about Trip. Searchers may find the alert function a bit basic and may prefer to use other tools for more sophisticated alerting needs.

## ANSWER ENGINE AND “BEST SUGGESTED” ANSWER

Trip mines content to provide links to answers that are a click or two away from the results screen [8]. For example, when searching for *naloxone*, Trip provides a prompt at the top of the results: “Trip has 9 answers for related questions.” Clicking the Show link provides a list of common topics; then another click takes the user to content on any of the following topics: naloxone and opioid-induced constipation, clinical pharmacology, indication and usage, contraindications, warnings, precautions, adverse reactions, overdosage, and dosage and administration. Answers are culled from reliable sources, such as the National Library of Medicine’s DailyMed.

Trip has several other methods for helping users find answers more quickly. The results of a search on “influenza vaccine pregnancy” include a box labeled, “Trip’s best suggested answer.” This box contains a short summary on the topic of vaccination benefits to the fetus and infant, followed by a link to a Canadian Pediatric Society statement. Users can also use the Clinical Q&A publication filter to identify synopses of their search topics. Synopses dates can vary widely from topic to topic, so some topics may not present results that are recent enough for clinical users.

## IMAGE AND VIDEO SEARCH

Search results can be limited to images and videos. Those results can be limited further to “Only show images that are free to modify, share and use,” making Trip a useful resource for presentations or training.

## FUTURE PLANS

Trip Database has announced that an automated review system will be available to Trip Pro users in January 2018 [9]. This tool will help users understand the results of multiple trials [10] and allow them to automatically generate a list of interventions from multiple trials with an estimate of likely effectiveness [11]. In the future, Trip will address the automated review of outcomes [12].

## PLATFORM

Trip is a web-based resource that is compatible with major browsers. A mobile version for all tablets and phone browsers works well for quick clinical searches [13]. The mobile version should be used in the portrait orientation for best viewing. No mobile app is available.

## COMPARISON TO OTHER PRODUCTS

The cost for Trip is modest compared to many point-of-care tools that are used in medical libraries. The search functions offer flexibility for naïve and sophisticated searchers. Trip’s search functions are less robust than most vendor implementations of MEDLINE but more robust than those found in most point-of-care resources. The unique capabilities of Trip’s results sorting are not found in other products. Specialized filters focus results and are valuable to those who are interested in evidence-based information for clinical decision-making. Trip also includes grey literature that is not available in many other tools.

## SUMMARY

Trip and Trip Pro offer many features of interest to clinicians and librarians by using ideas from linguistic analysis, answer summarization, crowdsourcing, and automation to provide quality information with fewer clicks. Trip combines results from many evidence-based sources, displaying them in an order that makes sense for evidence-based practice. Filters are designed with evidence-based clinical decision-making in mind. Increasing open access content and OpenURL linking to institutional journal collections puts full-text a click or two away from results. Output in RIS format allows users to import results into bibliographic citation databases. Alerts provide updates on Trip’s development along with links to new results on user topics.
